# Supplementary figures and images for: Integrative Analysis of the lncRNA and mRNA Transcriptome Revealed Genes and Pathways Potentially Involved in the Anther Abortion of Cotton (Gossypium hirsutum L.)
Source: Genes (Basel). 2019 Nov 20;10(12):947. doi: 10.3390/genes10120947 (PMC6947465; doi:10.3390/genes10120947)

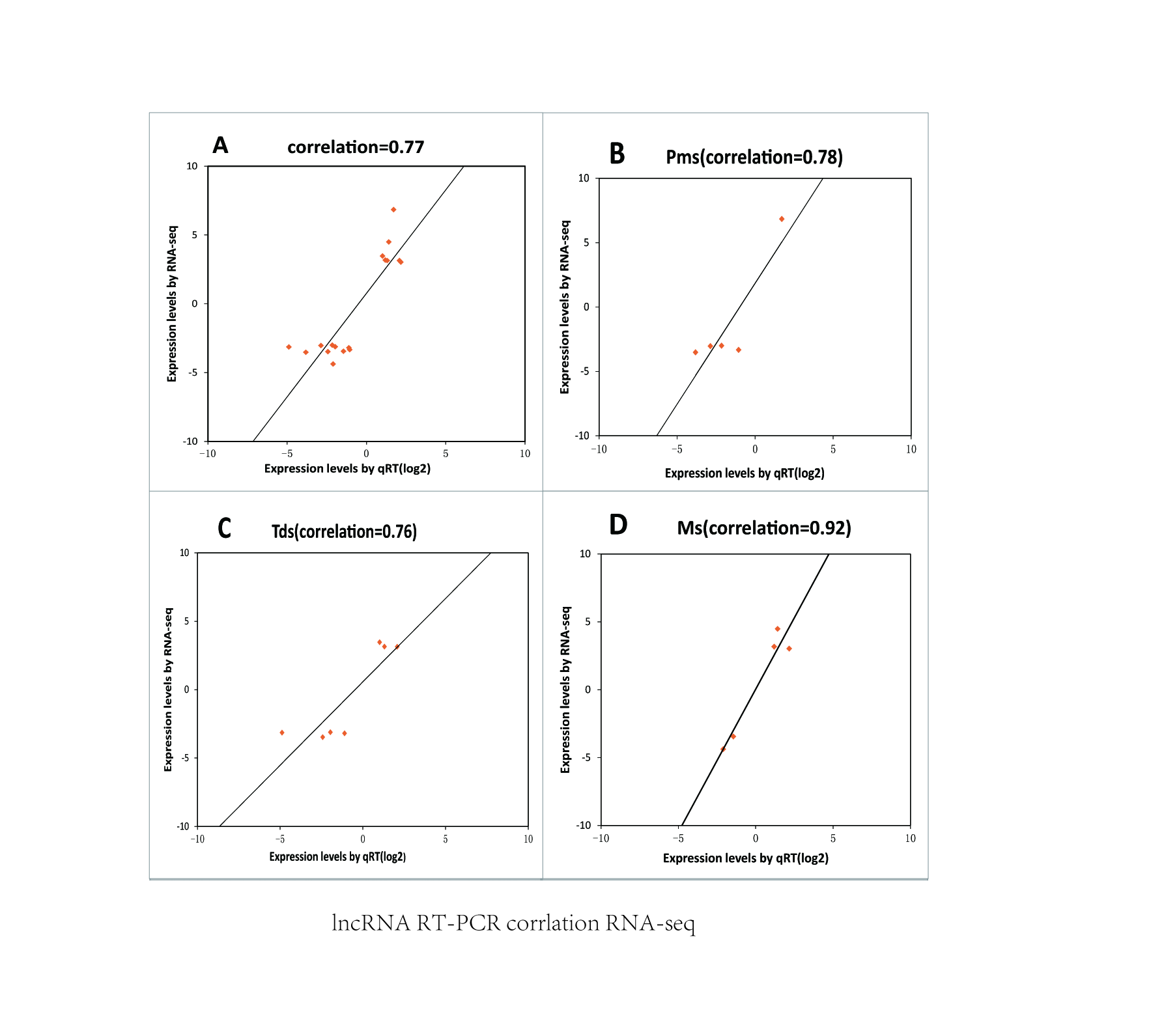

Supplement: Supplementary file 1 [file genes-10-00947-s001.zip › Supplementary/Figure S1. LncRNA RT-PCR correlation RNA-seq.tif]

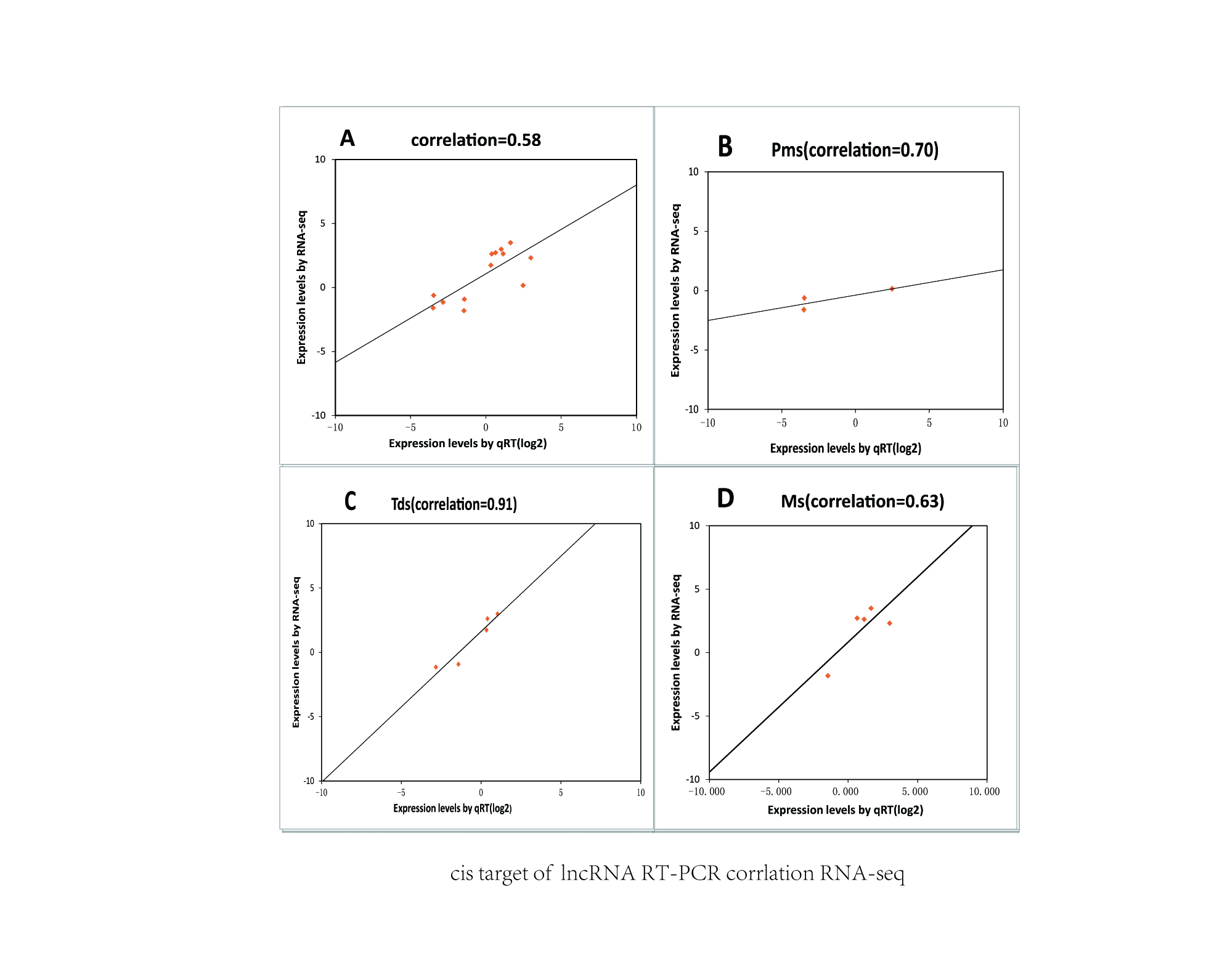

Supplement: Supplementary file 1 [file genes-10-00947-s001.zip › Supplementary/Figure S2. cis-target gene of lncRNA RT-PCR correlation RNA-seq.tif]
